# Study on Contact Stress Distribution Characteristics and Damage of Plug Seedlings Based on Flexible Pressure Sensor

**DOI:** 10.3390/s23198175

**Published:** 2023-09-29

**Authors:** Ji Cui, Xuying Li, Fandi Zeng, Hongbin Bai

**Affiliations:** College of Mechanical and Electrical Engineering, Inner Mongolia Agricultural University, Hohhot 010018, China; cuijiemail@163.com (J.C.); zfd19508@163.com (F.Z.); bhb81571@163.com (H.B.)

**Keywords:** plug seedlings, transplanting, damage, contact stress, parameter optimization

## Abstract

At present, there is a problem that the growth quality is reduced due to damage to the plug seedling pot during the transplanting process. In this study, the pressure distribution measurement system was used to measure the contact area of plug seedlings when they collided with the ground. The effects of seedling age and forward speed on the characteristics of contact stress distribution and potting damage were investigated through a single-factor experiment. The results were comprehensively considered based on the single-factor test, and the Box–Behnken test was used to optimize the design. The matrix loss rate was used as the evaluation index to determine the optimal parameter combination for transplanting: the tray specification was 72, the seedling age was 30 d, and the forward speed was 1.25 km·h^−1^. This study can provide a reference and technical support for further research on pot damage in plug seedling transplanting. The optimized parameters can provide practical guidance for reducing pot damage and improving growth quality during transplanting plug seedlings.

## 1. Introduction

As a modern cultivation technology, plug seedlings can shorten the fertility cycle of crops, improve crop yield, and ensure that crops are planted to meet agronomic requirements better [1]. Mechanized transplanting can reduce labor intensity and production costs, which is conducive to ensuring food security and is significant in building a powerful agricultural country and promoting rural revitalization [2,3,4]. The mechanized transplanting process will inevitably produce plug seedling pot damage; the damage to the pot is not only related to the material’s physical and mechanical properties but also to the impact load and the contact stress distribution during the transplanting process [5,6,7].

To reduce the pot damage of plug seedlings in the transplanting process, scholars have researched its mechanical properties and damage [8,9,10]. For example, Wen et al. [11] eliminate the seedling pot tumbling phenomenon in the seedling extraction process to avoid scattering lumps caused by the instantaneous impact of the top bar on the pot. Through the analysis of the mechanical model of the seedling pot in the extraction process, the factors affecting the rolling and damage of pot seedlings were obtained. Han [12] studied chili plug seedlings’ morphological characteristics, mechanical characteristics, and falling posture of pepper plug seedlings. Through the drop test, it is concluded that the water content and drop height of the seedling pot have an effect on the loss of the seedling pot matrix. Aiming at the low damage rate of the collision between the pot seedling and the cup mouth during the transplanting process, Wang et al. [13] established a collision contact mechanics model. Liu et al. [14] used Hertz’s theory and kinetic energy theorem to establish the mechanical equation of the collision between the seedling bowl and the planter. Then, they established a multi-objective optimization function by the objective programming method to obtain the optimal working parameters. In summary, many scholars have studied the damage of plug seedling pots through experiments and theoretical methods. However, the previous micro-analysis of collision damage to the plug seedling is mainly based on the Hertz theory. It did not consider the pot of the anisotropic deformation in the vicinity of the contact area. Therefore, there is an error in the calculation results. In recent years, with the development of high-performance data acquisition technology sensors, sensor technology has begun to be applied in the agricultural field [15,16,17]. Wu et al. [18] applied pressure sensing film to reveal the relationship between the contact pressure distribution and damage of the Korla pear, and preliminarily realized the prediction and evaluation of Korla pear damage. Piotr Komarnicki et al. [19] studied apples’ contact pressure and contact area when they collided with different materials through the Tekscan test system and proposed a bruise resistance index to determine the damage condition. Chen et al. [20] used a prescale induction film to measure the contact stress distribution characteristics and laws of potatoes when they collided with different materials at different drop heights and obtained a damage prediction model. At present, there is no relevant research on the distribution characteristics of contact stress and damage of plug seedlings in the process of mechanized transplanting.

Therefore, based on the pressure distribution measurement system, the contact stress distribution of the plug seedlings during the transplanting process was measured and studied. The contact stress distribution characteristics and the change rules of the damage to the pot during the collision of the plug seedlings under different factors during the transplanting process were studied. Using the matrix loss rate as a test index, three factors and three levels of orthogonal tests were used for the establishment of the regression model of the various factors and the matrix loss rate. Finally, the optimal parameter combination was obtained. This study is expected to provide a reference for the study of the damage of plug seedlings during transplanting.

## 2. Materials and Methods

### 2.1. Test Materials and Equipment

**Figure 1:**
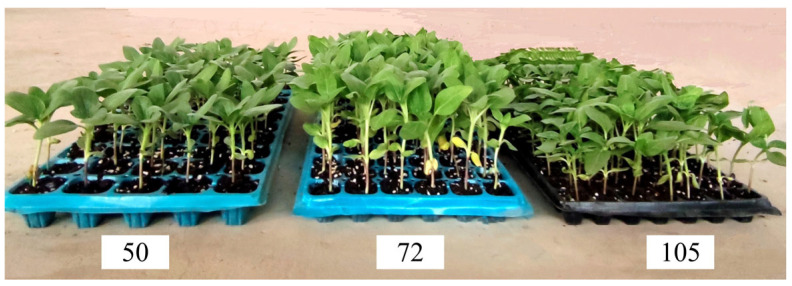
Different specifications of oil sunflower plug seedlings.

The plug seedlings used in the experiment were entrusted to Inner Mongolia Heyuan Agricultural Science and Technology Co., Ltd. for standardized cultivation. The size of the seedling plug was 540 mm × 280 mm. The T562 oil sunflower plug seedlings with plug specifications of 50, 72, and 105 holes were cultivated, respectively. The corresponding pot volumes were 60.27 cm^3^, 42.00 cm^3^, and 27.43 cm^3^, as shown in Figure 1. The plug seedling substrate was a combination of grass charcoal, vermiculite, and perlite, with a volume ratio of 3:1:1. The oil sunflower seedlings grew well, with no symptoms of pests and diseases and no apparent collapse phenomenon. Before the experiment, five matrix samples were randomly selected from each tray to determine the moisture content. The moisture content of the substrate was determined by using a DHG-9245A electric blast drying oven. The drying temperature was set to 105°, and the drying was conducted for 8 h. According to the formula of soil moisture content, the measured moisture content of the matrix ranged from 58% to 63% [21].

**Figure 2:**
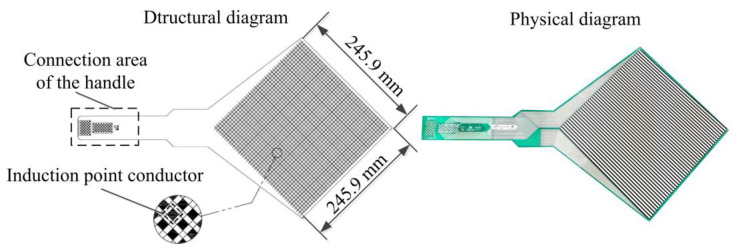
5250-model flexible film network tactile pressure sensor.

This study uses the Tekscan pressure distribution measurement system: an efficient, accurate, and intuitive tool. The working principle for the conductor cross-induction point array in the flexible film network is that when the tactile pressure sensor is subjected to external pressure, the resistance of the semiconductor will change proportionally with the change of the external force. The electrical signals of the sensing point of the conductor will be converted into real-time data. Through the I-Scan System data processing software, the data is used to image the data, display, and generate a two-dimensional or three-dimensional stress distribution map. The 5250-type flexible film network tactile pressure sensor is used in this test, as shown in Figure 2. It has a matrix-shaped sensing area (245.9 mm × 245.9 mm), a resolution of 3.2 cm^−2^, and a sampling frequency of 0–100 Hz [22,23]. The specific specifications are shown in Table 1.

**Figure 3:**
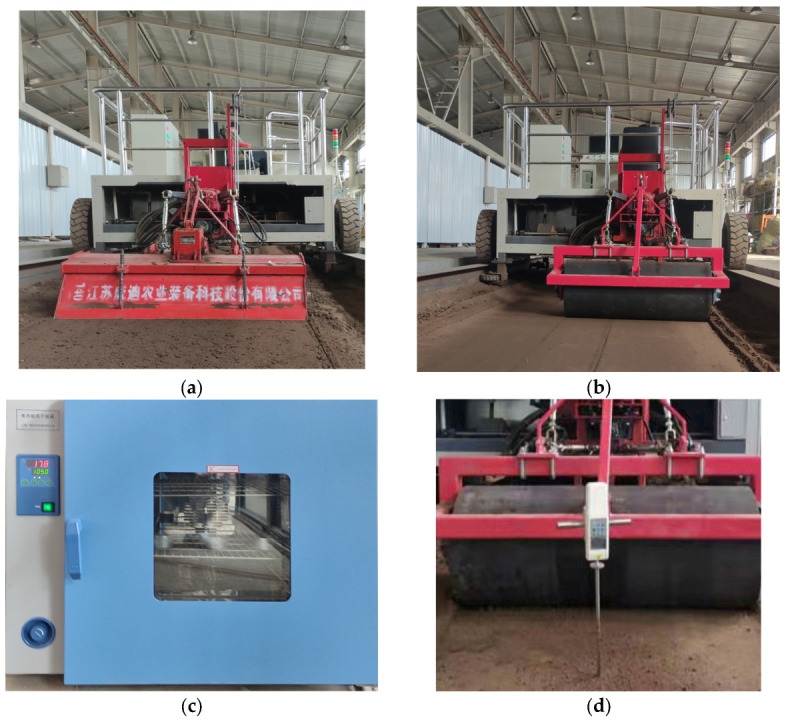
Soil pretreatment process: (**a**) rotary tillage; (**b**) soil compaction using a metal roller; (**c**) measurement of soil moisture content; (**d**) measurement of soil compactness.

The experiment was carried out in the soil trench laboratory of Inner Mongolia Agricultural University in Hohhot City, Inner Mongolia Autonomous Region. The soil type was sandy loam. In order to ensure that the soil conditions in the soil trough were in line with the transplanting operation, the soil was pretreated [24,25], as shown in Figure 3. First, the rotary cultivator is used for multiple rotary tillage, and the soil surface is entirely broken and evenly mixed. Then, the soil is sprayed with the spray system of the soil bin truck to adjust the soil moisture content, and then the compaction operation is carried out with the roller. The soil samples were collected by standard ring knife (diameter 52.5 mm, height 51 mm) at each point in the soil bin test area, and the soil samples were placed in a 105 °C drying oven. The soil moisture content determined by the drying method was 14.52 ± 1.33%. Five points in the test area were randomly selected to measure the soil compaction of 100 mm below the surface by using the TYD-2 soil compaction meter. The final determination of soil firmness was 97.5 ± 6.55 N·cm^−2^.

**Figure 4:**
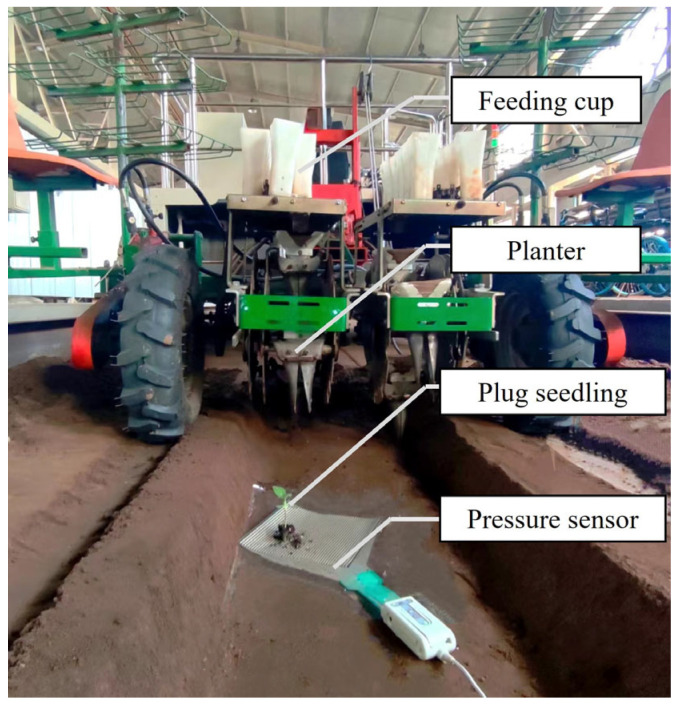
Transplanting touchdown collision test.

To protect the sensor and ensure consistent soil cushioning, a concave trench was dug manually in the hole-punching area of the planter. After adjusting the soil firmness, the pressure sensor was laid flat on the soil surface of the concave trench so that the planter’s lowest point and the pressure sensor’s surface were close. When starting the transplanting touchdown collision test, manually put the plug seedling into the seedling feeding cup. When the planter was rotated to the seedling receiving position, the bottom of the seedling feeding cup was opened, and the plug seedlings fell into the planter. When the plug seedling rotates to a certain position with the transplanter, the cup mouth opens, and the plug seedling falls onto the pressure sensors. The pressure distribution measurement system collects the contact information. Then, through the I-Scan System data processing system, the contact area and contact stress of the plug seedlings were obtained. The mass of the plug seedling was weighed before and after each transplanting to calculate the matrix loss rate of the plug seedling. After each test, the residual matrix on the surface of the sensor was cleaned to ensure the accuracy of the test. The study does not consider the transplanting machine ground wheel slip, mechanical vibration, and other factors. The transplanting touchdown collision test is shown in Figure 4.

### 2.2. Single-Factor Experimental Method

The plug seedling pot is a matrix-root complex, and the tightness of its growth condition is closely related to the tray specification and the age of the seedling. Therefore, referring to the relevant literature on seedling guidance, different specifications of the hole tray seedling should be selected for different seasons [26]. This experiment’s commonly used tray specifications were 50, 72, and 105. The standard seedling age of oil sunflower was 28–33 days, and the test seedling age was selected near the average seedling period of five days. According to the transplanting requirements of agricultural machinery and agronomy and related references [27,28], the forward speed of the transplanting machine was determined to be 1–2 km·h^−1^, and five levels were tested.

In order to obtain the orthogonal experiment factor level, take the tray specification of 50 plug seedlings as an example. The single-factor experiment of different seedling ages and forward speeds was carried out. The distribution characteristics of contact stress and the change rule of pot damage under different factors during transplanting were studied. The experiment was divided into two groups: (1) using a forward speed of 1.25 km·h^−1^ as a constant, carried out at different seedling ages for the single-factor experiment; (2) using a plug seedling age of 30 d as a constant, carried out at different forward speeds for the single-factor experiment. Each group of experiments was repeated 30 times, and the average value was the final value. The experimental factors and levels are shown in Table 2.

Refer to transplanting-related literature and agronomic requirements. The seedling stage is too long after transplanting due to the damage to the pot body and the root system. The matrix loss rate should be less than 15% to meet the transplanting conditions [29,30]. The percentage of matrix loss (*K*) characterizes the percentage of post-transplanted quality of plug seedlings compared to the substrate quality before transplanting [31], which is a criterion for judging the degree of damage to the pot. Therefore, the matrix loss rate was selected as the evaluation standard of planting quality to explore the variation rule of pot damage under different factors, as shown in the following equation:

(1) $$K=\frac{M-M_{1}}{M}\times 100\%$$

where *K* is the matrix loss rate, %; *M* is the mass of plug seedlings before transplanting, in grams; and *M*_1_ is the mass of plug seedlings after transplanting, in grams.

### 2.3. Box–Behnken Experimental Design

Based on the single-factor test, the level of orthogonal test factors was selected. The significant degree of the influence of seedling age, forward speed, and tray specification on the damage of plug seedling pot body during transplanting was studied. The experimental factors and level codes are shown in Table 3. The orthogonal test of three factors and three levels was conducted, and the matrix loss rate was the evaluation index. The regression model of seedling age, tray specification, and forward speed on the matrix loss rate was established. The optimal parameter combinations were finally obtained through significance analysis and interaction analysis, and their reliability was verified through experiments.

### 2.4. Data Processing

Excel 2021 was used for data entry, collation, and preliminary analysis. IBM SPSS Statistics 24 software was used for further research. One-way ANOVA and multiple comparisons (Duncan method, *p* < 0.05) were used to analyze the significant differences in the matrix loss rate and contact area of plug seedlings at different seedling ages and forward speeds. Origin 2021 was used for drawing.

## 3. Results

### 3.1. Rules of Pot Damage at Different Seedling Ages

**Figure 5:**
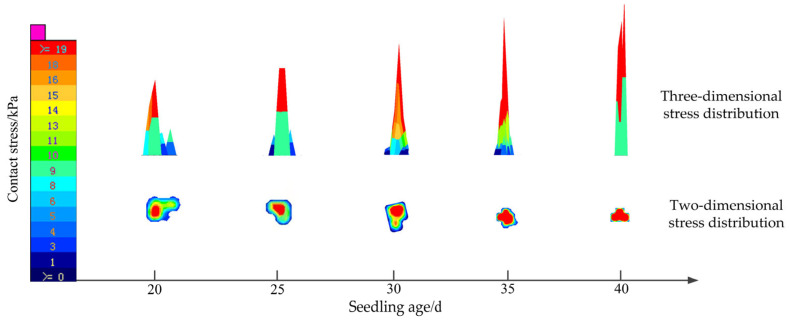
Relationship between seedling age and contact stress distribution.

With a forward speed of 1.25 km·h^−1^ and tray specification of 50, a touchdown collision test was carried out for plug seedlings of different seedling ages. The relationship between seedling age and the distribution of contact stress is shown in Figure 5. In the transplanting process, the plug seedling pot and the contact collision area occurred. The contact area gradually spread to the edge, and the maximum contact stress was located in the center of the contact area. With the increase of seedling age, the contact area of the plug seedling touching the ground collision area decreased. The area of high stress accounted for a more significant proportion, and the area of low stress accounted for a smaller proportion. The main reason is that when the seedling age is small, the root system of the plug seedling is poorly wrapped. The plug seedling pot is relatively loose and has poor cohesion, so the amount of deformation in the touchdown collision is large, and the contact area is extensive. With the increasing age of the plug seedling, the root system becomes developed, and the strength of the pot is gradually strengthened. The amount of deformation in the touchdown collision area decreases. The contact area is gradually reduced, accordingly.

**Figure 6:**
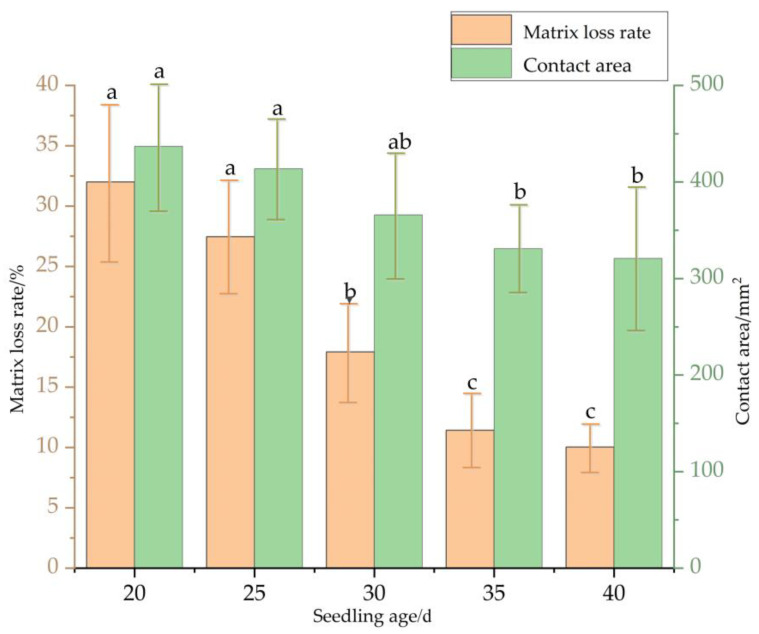
Relationship between seedling age and pot damage: the error bar is the standard mean value error; and different lowercase letters indicate that the data are statistically significant between the sample points.

The study continues by further investigating the changing rule of contact stress distribution when plug seedlings touch the ground during transplanting under different seedling ages. The test results are shown in Figure 6. In the study of the effect of seedling age on the collision of plug seedlings during transplanting, the forward speed and plug specifications are certain. With the increase of seedling age, the contact area decreased gradually. However, the matrix loss rate gradually decreased with the rise of seedling age.

When the seedling age was 30 days, relatively significant changes in contact area and matrix loss rate occurred and gradually became flat. The main reason is that from 35 days of age, the root coverage within a particular hole tray volume reaches the maximum. At this time, there is no noticeable increase in the degree of potting firmness. Accordingly, the contact area of the plug seedling does not vary much. The matrix loss rate of the plug seedling touching the ground collision area also gradually changes and tends to flatten out. In order to reduce the damage of plug seedlings during transplanting as much as possible, the matrix loss rate should be as low as possible to guarantee the survival rate and growth quality of the plug seedlings after transplanting. Therefore, a low matrix loss rate is shown at the seedling age of 25–35 days, which is the best time for transplanting.

### 3.2. Rules of Pot Damage at Different Forward Speeds

**Figure 7:**
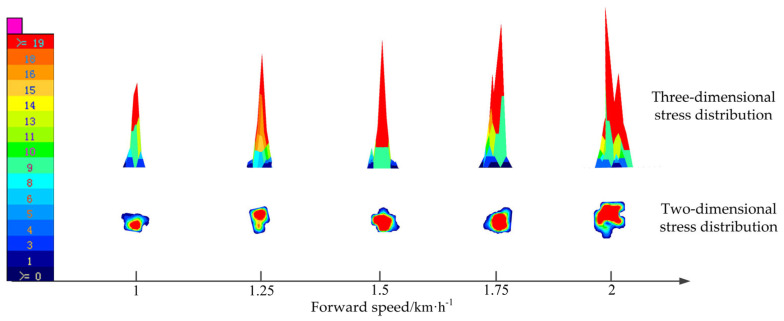
Relationship between forward speed and contact stress distribution.

With the seedling age of 30 d and a plug size of 50, the impact test of plug seedlings at different forward speeds was carried out. The relationship between forward speed and contact stress distribution is shown in Figure 7. It was found that the contact area and contact stress of the plug seedling touching the ground collision area increased with the increase of the forward speed of the transplanter. With the increase of forward speed, the distribution area of low stress was reduced and gradually concentrated towards the edge of the contact area. In contrast, the distribution area of high stress showed a trend of gradual expansion. In addition, the characteristic shape of the contact stress distribution contour of the plug seedling touching the ground collision area was close to an ellipse when the forward speed of the transplanter was low. However, when the forward speed of the transplanter was 2 km·h^−1^, the edge of the contact stress distribution contour of the plug seedling touching the ground collision area became irregular. The edges of the characteristic contour of contact stress distribution showed a certain degree of radial shape. These phenomena are because the kinetic energy of the seedling pot increases with forward speed, which leads to an increase in the amount of pot deformation when the seedling pot touches the ground. Therefore, the contact area and contact stress are increased. At the same time, the damage degree of the plug seedling pot was aggravated, and the scattered lump appeared.

**Figure 8:**
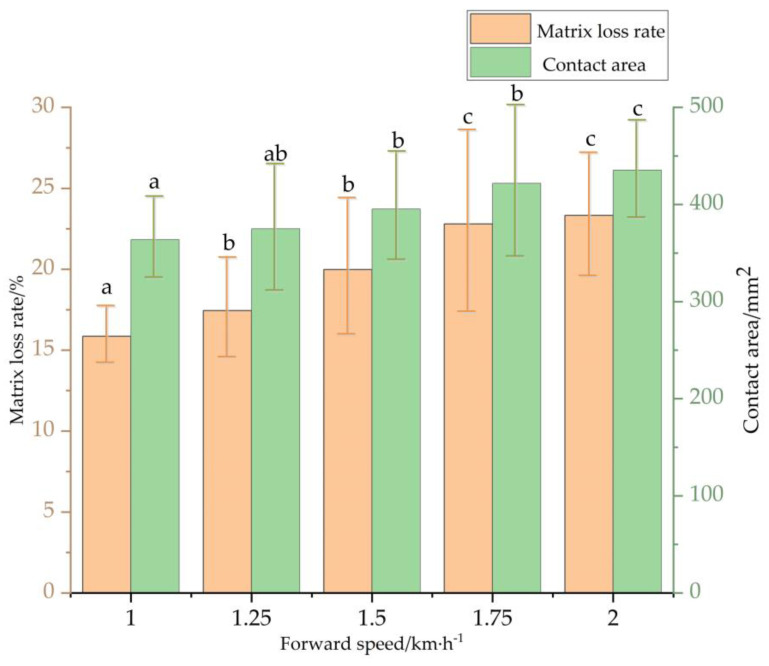
Relationship between forward speed and pot damage: the error bar is the standard error of the mean value; and different lowercase letters indicate that the data are statistically significant between the sample points.

The stress distribution characteristics and damage relationship of the plug seedlings pot at different forward speeds during transplanting were further investigated, as shown in Figure 8. When the seedling age and tray specifications are sure, with the increase of forward speed, the contact area and the matrix loss rate of the plug seedling when touching the ground collision are gradually increasing. This is because the strength and stability of the root-substrate complex of plug seedlings are relatively particular at a certain seedling age and tray specification. When the forward speed increases, the kinetic energy of the seedling gradually increases. Additionally, the amount of deformation of the seedling complex under stress increases, the contact area increases, and the matrix loss rate of the seedling increases.

When the forward speed is *v* = 1.75 km·h^−1^, the matrix loss rate and contact area inflection point tend to be gentle; this shows that the plug seedlings have greater kinetic energy currently. When the plug seedling touches the ground, it produces a large impact force and excessive load, and its pot undergoes severe deformation. As the speed increases, many nursery substrate particles slip and collapse due to excessive force. This results in more severe matrix damage and visible deformation of the plug seedling pot. The matrix loss rate has also reached the maximum, and the contact area during ground collision has reached more than 430 mm^2^. Therefore, to ensure that the damage to plug seedlings is small, a forward speed is selected in the 1–1.5 km·h^−1^ range.

### 3.3. Box–Behnken Experimental Optimization Design

This experiment considered the effects of different factors (seedling age, tray specification, and forward speed) on the matrix loss rate during plug seedling transplanting. The design factors included different levels of seedling age (25, 30, and 35 days), tray specification (50, 72, and 105), and forward speed (1, 1.25, and 1.5 km·h^−1^). A total of 17 groups of experiments were designed by Box–Behnken experimental design, coded according to high (+1) and low (−1) levels, and analyzed in Design Expert software. The experimental design and results are shown in Table 4.

The multiple regression analysis of the test results in Table 4 was carried out by Design-Expert software, and the second-order regression model of the matrix damage rate and three significant parameters was obtained. The equation is as follows:

(2) $$K=13.10-4.54X_{1}+1.59X_{2}-3.72X_{3}-0.60X_{1}X_{2}+2.36X_{1}X_{3}-0.57X_{2}X_{3}+0.17X_{1}{}^{2}+0.28X_{2}{}^{2}+1.37X_{3}{}^{2}$$

The analysis of the variance of the regression model of the Box–Behnken experimental design is shown in Table 5. The *p* < 0.0001 of the fitting model, the determination coefficient R^2^ = 0.9781, and the corrected determination coefficient R^2^_adj_ = 0.9499 are close to 1, indicating that the fitting equation is meaningful and its credibility is high. The signal-to-noise ratio (Adeq precision) is 21.720, indicating that the regression model is highly significant. The loss of fit term *p* = 0.1562 > 0.05 indicates that the regression equation is still intact and is well-fitted. Seedling age *X*_1_, forward speed *X*_2_, tray specification *X*_3_, and interaction term *X*_1_ *X*_3_ significantly affected the matrix loss rate. *X*_1_ *X*_2_, *X*_2_ *X*_3_, *X*_1_^2^, *X*_2_^2^, and *X*_3_^2^ did not significantly affect the matrix loss rate.

Therefore, excluding the factors that have no significant effect on the quadratic regression model, the optimized regression equation is the following:

(3) $$K_{1}=13.54-4.54X_{1}+1.59X_{2}-3.72X_{3}+2.37X_{1}X_{3}$$

### 3.4. Analysis of Interaction

**Figure 9:**
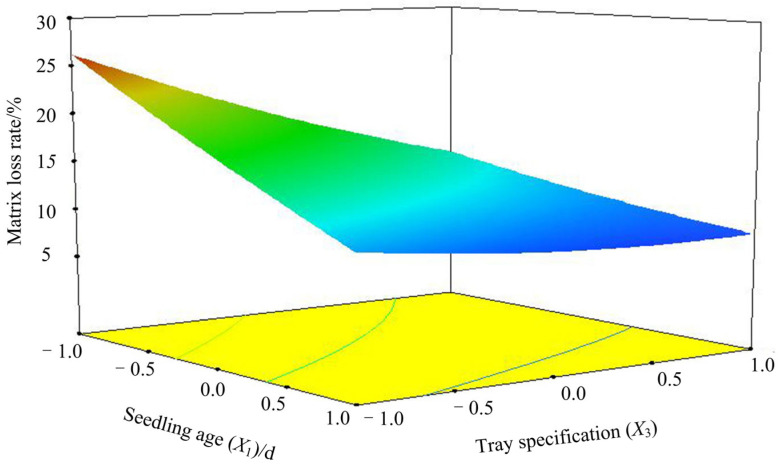
*X*_2_ = 1.25 km·h^−1^; *X*_1_ *X*_3_ interaction response surface plot.

The response surface analysis diagram and the corresponding contour map of different factors were obtained by Design-Expert software, as shown in Figure 9. The response surface graph is a three-dimensional space surface graph of the response value matrix loss rate to the test factors of seedling age and tray specification. From the response surface analysis diagram, it can be visually seen that the interaction of seedling age and tray specification factors affects the matrix loss rate. If the curve is steeper, it indicates that the influence of this factor on the matrix loss rate is greater, and the corresponding performance is the size of the response value change. It can be seen from the contour map that the greater the curvature of the contour line, the more significant the interaction between the two parameters. The response surface software and regression equation were used to analyze the interaction of different factors on the response value. When the forward speed was intermediate (1.25 km·h^−1^) and the seedling age was constant, the matrix loss rate showed a downward trend when the plug size ranged from 50 to 105. This occurs because as the tray specification becomes larger (with more holes), the volume of each hole is smaller, and the volume of the plug seedling pot is smaller. At this time, the root coverage rate is significant, and the gathering effect is obvious. Accordingly, the pot damage is small; when the tray specification is constant, the matrix loss rate decreases with the increase of seedling age. This occurs because, with the increase of seedling age, the root system of plug seedlings absorbs the nutrients in the matrix. The more developed the root system, the better the encapsulation and compactness. Therefore, the pot of the plug seedling pot is less damaged during the transplanting process.

### 3.5. Determination of the Optimal Parameter Combination

According to the test results, the optimization module in Design-Expert software was used to determine the optimal value and the range of influencing factors. The regression model (2) was optimized, and the working characteristics of the transplanting process were comprehensively considered. The expectation analysis revealed the optimal parameter combination: the tray specification was 72, the seedling age was 30 d, and the forward speed was 1.25 km·h^−1^. Under these conditions, the matrix loss rate was 13.50%.

## 4. Discussion

This study aims to analyze the stress distribution characteristics of plug seedlings and the damage rules of pots under different plug specifications, seedling ages, and forward speeds during transplanting and to explore the relationship between them. The experiments were carried out with the help of a flexible pressure sensor, and the damage law of the pot in the transplanting process was accurately revealed.

The results showed that with the increase in seedling age, the pot damage decreased, which was consistent with the trend shown in the literature [28], but there was a small difference. The reason may be that the test scheme is different. In the previous study, the transplanter did not have a forward speed, and the height of the bottom of the planter from the ground was too high. The type of plug seedlings, matrix ratio, and moisture content will also affect the pot damage.

From the point of view of the research on the damage of the pot by the forward speed, the forward speed of the transplanter increases, and the rotation speed of the planter increases. The relative speed of the plug seedling to the ground increases when the seedling touches the ground. Some studies mainly focus on the collision damage of the plug seedling pot caused by the rotation of the planter. It is concluded that with the increase in the planter’s rotation speed, the pot and the planter’s relative speed increases, and the maximum collision force becomes larger [13,26].

Several factors are related to tray specification, such as the volume of the pot, the influence of the contact stress distribution, and the pot damage. We previously obtained through the vertical drop test that, with the increase of the tray specification, the contact stress area and the pot damage were reduced when the plug seedlings collided with the ground [22,32]. Although the posture and number of collisions of plug seedlings under actual transplanting conditions are variable, it is consistent with the rules obtained under the previous vertical drop conditions, so we only elaborated a little.

The current research combines the actual working conditions of transplanting, the relationship between the stress distribution characteristics, and the damage of plug seedlings during transplanting. However, the transplanter did not plant plug seedlings into the soil. Therefore, the next step will continue to explore the stress distribution characteristics of plug seedlings and the changing rule of pot damage during the process of soil entry to improve the efficiency and quality of the transplanting technology of the plug seedling transplanter.

## 5. Conclusions

In this study, the oil sunflower plug seedlings were taken as the research object. Based on the deep integration of agricultural machinery and agronomy, a pressure distribution measurement system was used to conduct the collision test of plug seedlings when transplanting. The variation rule of pot damage and contact stress distribution of plug seedlings under different factors in the transplanting process was obtained. Considering the test results comprehensively, the Box–Behnken test optimization design obtained the optimal parameter combination. The following are the main conclusions:

1. When the forward speed and plug specifications were constant, the contact stress increased gradually with the increase of seedling age, but the contact area and matrix loss rate decreased gradually. When the seedling age and soil plug specifications are constant, the contact stress, contact area, and matrix loss rate gradually increase with the forward speed.
2. This study established the regression model of the three factors on the matrix loss rate by the orthogonal test of seedling age, tray specification, and forward speed. The results showed that the tray specification, seedling age, and forward speed had highly significant effects on the matrix loss rate, and there was an interaction between seedling age and tray specification.
3. Through the Design Expert software, the optimal parameter combination is the following: the tray specification is 72, the seedling age is 30 d, and the forward speed of the transplanter is 1.25 km·h^−1^. The repeated experiments show that the parameter combination is reliable and can be used in plug seedling transplanting production operations.

## Figures and Tables

**Table 1:** The specifications of the pressure sensor.

Items	Specifications
Number of Sensels	1936
Pitch	5.588 mm
Thickness	0.102 mm
Max Pressure	1724 kPa

**Table 2:** Single-factor test factors and levels.

Factors	Level				
	1	2	3	4	5
Seedling age/d	20	25	30	35	40
Forward speed (*v*)/km·h^−1^	1	1.25	1.5	1.75	2

**Table 3:** Influencing factors and levels.

Level	Factors		
	Seedling Age (*X*_1_)/d	Forward Speed (*X*_2_)/km·h^−1^	Tray Specification (*X*_3_)
−1	25	1	50
0	30	1.25	72
1	35	1.5	105

**Table 4:** Box–Behnken experimental design and results.

Test Number	Seedling Age (*X*_1_)/d	Forward Speed (*X*_2_)/km·h^−1^	Tray Specification (*X*_3_)	Matrix Loss Rate/%
1	−1	0	−1	27.49
2	−1	0	1	13.05
3	0	0	0	13.56
4	1	−1	0	8.33
5	0	1	−1	19.98
6	0	0	0	13.86
7	1	0	−1	11.13
8	0	1	1	12.01
9	1	1	0	10.36
10	0	0	0	13.86
11	0	−1	−1	15.00
12	0	0	0	13.86
13	1	0	1	7.51
14	−1	−1	0	16.27
15	0	−1	1	10.88
16	−1	1	0	21.00
17	0	0	0	13.86

**Table 5:** Variation analysis of Box–Behnken design quadratic model.

Source of Variation	Mean Square	Degree of Freedom	Sum of Square	*p*-Value
Model	378.24	9	42.03	<0.0001 **
*X*_1_	204.83	1	204.83	<0.0001 **
*X*_2_	20.70	1	20.70	0.0044 **
*X*_3_	113.63	1	113.63	<0.0001 **
*X*_1_ *X*_2_	1.82	1	1.82	0.2596
*X*_1_ *X*_3_	29.27	1	29.27	0.0017 **
*X*_2_ *X*_3_	3.71	1	3.71	0.1237
*X*_1_^2^	0.70	1	0.70	0.4709
*X*_2_^2^	0.03	1	0.03	0.8839
*X_3_^2^*	3.31	1	3.31	0.1424
Residual	8.48	7	1.21	
Lack of fit	5.89	3	1.96	0.1562
Pure error	2.59	4	0.65	
Sum	386.72	16	42.03	

Note: ** indicates extremely significant (*p* < 0.01).

## Data Availability

Not applicable.

## References

[1] Yan Z., Wang L., Wang Y., Chu Y., Lin D., Yang Y. (2021). Morphological and Physiological Properties of Greenhouse-Grown Cucumber Seedlings as Influenced by Supplementary Light-Emitting Diodes with Same Daily Light Integral. Horticulturae.

[2] Wei H., Ye X., Du Z., Fan S., Robilian, Liu S., Huang C. (2022). Accelerate the construction of a new development pattern and focus on promoting the high-quality development of agriculture and rural areas—Authoritative experts deeply interpret the spirit of the 20th National Congress of the Communist Party of China. Chin. Rural. Econ..

[3] Zhao K. (2014). Research on the impact of agricultural mechanization development on the transformation of China’s agricultural economic development mode. Ph.D. Thesis.

[4] Zhiwei T., Wei M., Qichang Y., Sen Y., Mei Z., Famin D., Haidong X. (2022). Research status and problem analysis of greenhouse cave tray seedling transplanting machinery. J. China Agric. Univ..

[5] Dihingia P.C., Kumar G.P., Sarma P.K., Neog P. (2017). Production of soil block seedlings in plug trays for mechanical transplanting. Int. J. Veg. Sci..

[6] Han L., Kumi F., Mao H., Hu J. (2019). Design and tests of a multi-pin flexible seedling pick-up gripper for automatic transplanting. Appl. Eng. Agric..

[7] Jorg O.J., Sportelli M., Fontanelli M., Frasconi C., Raffaelli M., Fantoni G. (2021). Design, development and testing of feeding grippers for vegetable plug transplanters. AgriEngineering.

[8] Mohamed S., Liu J. (2019). Effect of Soil Moisture Content and End-Effector Speed on Pick-up Force and Lump Damage for Seedling Transplanting. AgriEngineering.

[9] Peng W., Xiuhua Z., Fukun H., Shuo H., Maokai J. (2021). Design and simulation of taking-putting seedling manipulator of plug seedling transplanter. Proceedings of the 2021 ASABE Annual International Virtual Meeting, 2021.

[10] Wang M., Song J., Liu C., Wang Y., Sun Y. (2015). Design and test of crank pendulum type seedling clamping mechanism of vegetable transplanting machine. Trans. Chin. Soc. Agric. Eng..

[11] Wen Y., Zhang J., Zhang Y., Tian J., Yuan T., Tan Y., Li W. (2020). Development of vegetable burrow tray seedling insertion ejection type seedling retrieval device. Trans. Chin. Soc. Agric. Eng..

[12] Han C. (2014). Design and Experimental Study of Automatic Feeding System of Cave Tray Seedling Transplanting Machine. Ph.D. Thesis.

[13] Wang Y., Chen J., Wu J., Zhao Y. (2014). Mechanical properties test of broccoli bowl seedlings for mechanized cultivation. Trans. Chin. Soc. Agric. Eng..

[14] Liu Y., Mao H., Wang T., Li B., Li Y. (2018). Optimization and experiment of movement analysis and experiment of tomato cave tray seedlings in hanging cup transplanting mechanism. Trans. Chin. Soc. Agric. Mach..

[15] Holthusen D., Brandt A.A., Reichert J.M., Horn R., Fleige H., Zink A. (2018). Soil functions and in situ stress distribution in subtropical soils as affected by land use, vehicle type, tire inflation pressure and plant residue removal. Soil Tillage Res..

[16] Sun N., Fan B., Ding Y., Liu Y., Bi Y., Seglah P.A., Gao C.J.S. (2023). Analysis of the Development Status and Prospect of China’s Agricultural Sensor Market under Smart Agriculture. Sensors.

[17] Wang X., Hu H., Wang Q., Li H., He J., Chen W. (2017). Discrete-based soil model parameter calibration method. Trans. Chin. Soc. Agric. Mach..

[18] Wu J., Guo K., Gu R., Zhang J., Qi C. (2010). Dynamic viscoelastic characteristics of Korla pears at different expansion levels. Trans. Chin. Soc. Agric. Mach..

[19] Komarnicki P., Stopa R., Kuta Ł., Szyjewicz D. (2017). Determination of apple bruise resistance based on the surface pressure and contact area measurements under impact loads. Comput. Electron. Agric..

[20] Chen Z., Duan H., Cai X., Wang J., Xu T., Yu g., Yao F., Yan F. (2020). Contact stress distribution characteristics of potato drop collisions. J. South China Agric. Univ..

[21] Wang Y., Yu H. (2015). Experiment and analysis of influencing factors of substrate integrity rate of seedling retrieval by manipulator of acupuncture tray seedlings. Trans. Chin. Soc. Agric. Eng..

[22] Bai H., Li X., Zeng F., Cui J., Zhang Y. (2022). Study on the Impact Damage Characteristics of Transplanting Seedlings Based on Pressure Distribution Measurement System. Horticulturae.

[23] Cui J., Li X., Zeng F., Bai H., Zhang Y.J.A.S. (2023). Parameter Calibration and Optimization of a Discrete Element Model of Plug Seedling Pots Based on a Collision Impact Force. Appl. Sci..

[24] He C., Guo Y., Guo X., Sang H. (2023). A mathematical model for predicting the draft force of shank-type tillage tine in a compacted sandy loam. Soil Tillage Res..

[25] Yan D. (2021). Simulation analysis and experimental study of soybean seed particle modeling, seeding and soil covering suppression process. Ph.D. Thesis.

[26] Liu Y. (2019). Optimization and Experiment of Vegetable Hole Tray Seedling Transplanting Seedling Bowl Breakage Mechanism and Planter Hole Forming Movement. Ph.D. Thesis.

[27] Fandi Z., Xuying L., Hongbin B., Ji C., Xuening L., Yongzhi Z. (2023). Experimental Research and Analysis of Soil Disturbance Behavior during the Hole Drilling Process of a Hanging-Cup Transplanter by DEM. Processes.

[28] Tang H. (2016). Parameter Optimization and Performance Experimental Study of Vegetable Bowl Seedling Hanging Cup Planter. Master’s Thesis.

[29] He Y. (2018). Design and Research of Automatic Seedling Retrieval System for High-Speed Planting of Vegetable Burrow Seedlings. Ph.D. Thesis.

[30] Zhang J., Long X., Han C., Yuan P., Gao J. (2021). Design and test of mechanical driven pepper burrow tray seedling automatic seedling taking and feeding system. Trans. Chin. Soc. Agric. Eng..

[31] Han B., Shen D., Guo C., Liu Q., Wang X., Song C. (2019). Design and test of adjustable end effector for seedling extraction of cabbage bowls. Trans. Chin. Soc. Agric. Mach..

[32] Zeng F., Li X., Bai H., Cui J., Liu X., Zhang Y. (2022). Experimental Study on Pot Damage and Contact Stress Distribution Characteristics of Oil Sunflower Plug Seedlings. Appl. Sci..

